# A Systematic Evaluation of Mobile Health Applications for the Prevention of Suicidal Behavior or Non-suicidal Self-injury

**DOI:** 10.3389/fdgth.2021.689692

**Published:** 2021-07-26

**Authors:** Lasse B. Sander, Marie-Luise Lemor, Racine J. A. Van der Sloot, Eva De Jaegere, Rebekka Büscher, Eva-Maria Messner, Harald Baumeister, Yannik Terhorst

**Affiliations:** ^1^Department of Rehabilitation Psychology and Psychotherapy, Institute of Psychology, Albert-Ludwigs-University of Freiburg, Freiburg, Germany; ^2^Department of Head and Skin, Flemish Centre of Expertise in Suicide Prevention, Ghent University, Ghent, Belgium; ^3^Department of Clinical Psychology and Psychotherapy, Institute of Psychology and Education, Ulm University, Ulm, Germany

**Keywords:** suicidal behavior, non-suicidal self-injury, mobile health apps, e-health, prevention

## Abstract

People with suicidal ideation and non-suicidal self-injury (NSSI) behavior face numerous barriers to help-seeking, which worsened during the COVID-19 pandemic. Mobile health applications (MHA) are discussed as one solution to improve healthcare. However, the commercial app markets are growing unregulated and rapidly, leading to an inscrutable market. This study evaluates the quality, features, functions, and prevention strategies of MHA for people with suicidal ideation and NSSI. An automatic search engine identified MHA for suicidal behavior and NSSI in the European commercial app stores. MHA quality and general characteristics were assessed using the Mobile Application Rating Scale (MARS). MHA of high quality (top 25%) were examined in detail and checked for consistency with established suicide prevention strategies. Of 10,274 identified apps, 179 MHA met the predefined inclusion criteria. Average MHA quality was moderate (M = 3.56, SD = 0.40*)*. Most MHA provided emergency contact, but lacked security features. High-quality MHA were broadly consistent with the best-practice guidelines. The search revealed apps containing potentially harmful and triggering content, and no randomized controlled trial of any included MHA was found. Despite a large heterogeneity in the quality of MHA, high-quality MHA for suicidal behavior and NSSI are available in European commercial app stores. However, a lack of a scientific evidence base poses potential threats to users.

## Introduction

Suicidal behavior is a major global health challenge with more than 800,000 persons dying by suicide every year, an even higher amount of suicide attempts, and a major burden caused by suicide bereavement (1, 2). The coronavirus disease 2019 (COVID-19) pandemic is discussed to be associated with a further increased risk of suicidal ideation, behavior, und death (3–5). Indeed, the current pandemic has already led to a massive increase in posttraumatic stress symptoms (6) and increased risk of depression (7) in patients with COVID-19, and worsening of mental health symptoms in some patients with preexisting mental disorders (8, 9). These conditions are highly associated with an elevated risk of suicidal behavior (10, 11). Furthermore, the harsh restrictions on individual movement and social interaction, intended to reduce the incidence of infections, lead to psychological distress in the general population (12). Thus, based on established intention-to-action models in suicide research, these restrictions may lead to further increases in suicide rates (13–16), while simultaneously impeding access to mental health services, making face-to-face meetings between therapists and patients very difficult or even impossible (13, 17). This is of particular concern as persons with suicidal ideation already show low help-seeking behavior (18, 19) and calls for innovative remote support measures.

Because smartphones have become omnipresent, mobile health applications (MHA) have the potential to increase the access to evidence-based support by overcoming some barriers of traditional mental health treatment for people with suicidal ideation, such as stigmatization, perception that professional treatment is not needed, or lack of time in an acute suicidal crisis (18). Thereby, MHA can provide help promptly, conveniently, and discreetly at low cost (20), especially in an acute crisis as they are independent of time or place (21).

To enfold this potential for suicide prevention, MHA should include methods to reduce or eliminate risk factors for suicidal behavior. Despite interventions directly targeting to reduce suicidal behavior or facilitating access to crisis support, non-suicidal self-injury (NSSI) has repeatedly been discussed as a major risk factor for suicidal behavior (22). In fact, NSSI has been incorporated into several models that seek to explain the development of suicidal behavior (23), including the integrated motivational–volitional model of suicidal behavior (24).

MHA for NSSI and the prevention of suicidal behavior are, however, particularly prone to risks for users, due to unnoticed harmful information, frustration due to lack of functionality, or recommendations that are not guideline-directed (25–27).

In a previous systematic assessment of smartphone tools for suicide prevention, Larsen et al. (20) reviewed the content of 49 apps referring to suicide or deliberate self-harm from the Australian Android and Apple App stores in 2015 (20). They found that most apps focused on one single suicide prevention strategy (e.g., obtaining support from friends and family) instead of providing comprehensive evidence-based support.

Given the rapid developments in the app market, this study presents an up-to-date overview of the quality, functions, and features of MHA for prevention of suicidal behavior and NSSI.

The aim of this study is to provide an overview of general characteristics, a standardized quality rating, and content analysis of MHA for the prevention of suicidal behavior and NSSI in European commercial app stores (iOS Store and Google Play Store). The following questions were addressed:

1. What is the quality of MHA or the prevention of suicidal behavior and NSSI in European commercial app stores regarding user involvement, functionality, aesthetics, and quality of information?
2. Which features, functions, and suicide/NSSI prevention strategies do the best rated MHA include?
3. Do the used features, functions, and suicide/NSSI prevention strategies mirror the “best practice” for the treatment of NSSI/suicidal behavior?

## Methods

### Search Strategy and Selection Procedure

Suicidal behavior and NSSI-related search terms were identified according to the respective relevant literature (for the complete search term, see Appendix A). The chosen search terms were used to screen the European Apple App Store and Google Play Store systematically for potentially relevant MHA using the automatic search engine of the Mobile Health App Database (MHAD; http://mhad.science) project (28), whose functionality and validity have been proven in previous studies (29–31). The search was conducted in August 2020 with equivalent search terms for suicidal thoughts and behavior in German, English, Dutch, and Spanish languages and for NSSI in German and English languages.

### Inclusion and Exclusion Criteria

All apps identified by the search engine were documented (automated extraction of e.g., title, version, download link, and app description), and duplicates were removed automatically. The eligibility of the identified apps was assessed in a two-step process: first, the description and pictures of the identified MHA were screened, whether the MHA was (a) designed for the treatment of suicidal/NSSI behavior and/or suicide/NSSI prevention and (b) available in one of the aforementioned languages. In a second step, the remaining MHA were considered eligible for the assessment if they additionally met the following criteria: (c) the MHA functions well enough to allow downloading and assessment and (d) the access of the content of the MHA requires no further gadgets or information (e.g., zip code or institutional login information).

### Data Extraction, Evaluation Criteria, and Instruments

Two independent reviewers (psychologists supervised by a licensed psychotherapist who reconfirmed all final ratings) evaluated each MHA and extracted all data using the German or English version of the Mobile Application Rating Scale (MARS) (32, 33). The MARS is a reliable and valid scale to assess app quality (34). The MARS is divided into a classification section, a quality rating section, and three additional subjective subscales.

In advance to the rating process, all reviewers underwent a standardized online reviewer-training provided by the developers. To test for accuracy of the ratings, the Intraclass correlation (ICC) between reviewers of the same MHA was calculated (35). An ICC > 0.75 was considered as an indicator for a satisfactory reviewer agreement (36). In case of a low reviewer agreement (ICC < 0.75), the rating was reviewed, and a third reviewer (LS) was consulted. For all analyses, the ratings of both reviewers were averaged.

### General MHA Characteristics: Classification Section

For the present work, the classification section of the MARS was modified to cover the following dimensions: (a) app name and URL, (b) platform, (c) affiliation (d) obligatory payment, (e) involvement in therapy, (f) security and privacy, (g) user rating, (h) technical features, (i) certification, and (j) emergency contact.

### Quality Rating

The quality rating of the MARS consists of 19 items, with a scale ranging from one (inappropriate) to five (excellent) (32, 33). The items are distributed among four objective subscales: (a) user engagement (five items: entertainment, interest, customization, interactivity, and target group), (b) functionality (four items: performance, usability, navigation, and gestural design), (c) aesthetics (three items: layout, graphics, and visual appeal), and (d) information quality (seven items: accuracy of app description, goals, quality of information, quantity of information, quality of visual information, credibility, and evidence base). For the evaluation of the overall MHA quality, the total score was determined from the four objective subscales (33). Three additional subscales, not impacting the mean scores, were also assessed: (e) therapeutic usefulness, (f) subjective quality, and (g) perceived impact. Means and standard deviations were calculated for each subscale and the overall quality score.

### User Star Rating

The star ratings (one to five stars) from users were extracted from the app stores. Bivariate correlations between the user ratings and the MARS overall rating score were investigated. Only user ratings with a minimum of three user ratings were included in the analyses.

### Evidence

To identify potential evidence on the feasibility or effectiveness of included apps, we conducted systematic literature searches in PubMed and PsycINFO. The searches were performed in March 2021. The search string comprised the names of all suicide/NSSI MHA identified in the app store searches and additional terms on suicidal behavior and self-harm, combined with terms on mobile apps (see Appendix B). In a first step, we screened titles and abstracts for studies on MHA for suicide/NSSI. In a second step, we screened full articles for inclusion, and it was checked whether the studies reported on apps that were identified in the app store searches.

### Features, Functions, and Prevention Strategies of High-Quality MHA

To describe and evaluate specific MHA features, functions, and suicide/NSSI prevention strategies of high-quality MHA, MHA in the top quartile of rating scores of all included MHA were examined in greater detail and compared with established suicide prevention strategies.

Therefore, the following information was captured: target group (persons affected by suicide/NSSI), their affiliated environment, healthcare professionals (like psychotherapists), certification (awards or certificates), and the provision by a credible source [competitive government or research funding, governmental/university agencies, non-governmental organizations (NGOs)/institutions, or specialized commercial companies/funding agencies].

Suicide prevention strategies were reviewed and categorized according to a modified classification scheme presented by Larsen et al. (20), which itself was derived from prior synopses (37, 38). For this study, the classification scheme was extended by the category of the main purpose of the MHA [provision of information, (emergency) resources, and/or urge/behavior management strategies/tools]. High-quality MHA were classified according to their purpose, screening strategies (physician- or self-screening), accessing support strategies (peer and family support, non-crisis support, crisis support, and visibility at all times), and mental health/treatment strategies (psychotherapy, safety plan, limiting access to means, identification of warning signs, identification of triggers, and coping strategies). We handled MHA for suicide prevention and MHA for NSSI prevention as belonging to one pathology and accordingly categorized in the same classification scheme.

## Results

### Search

**Figure 1:**
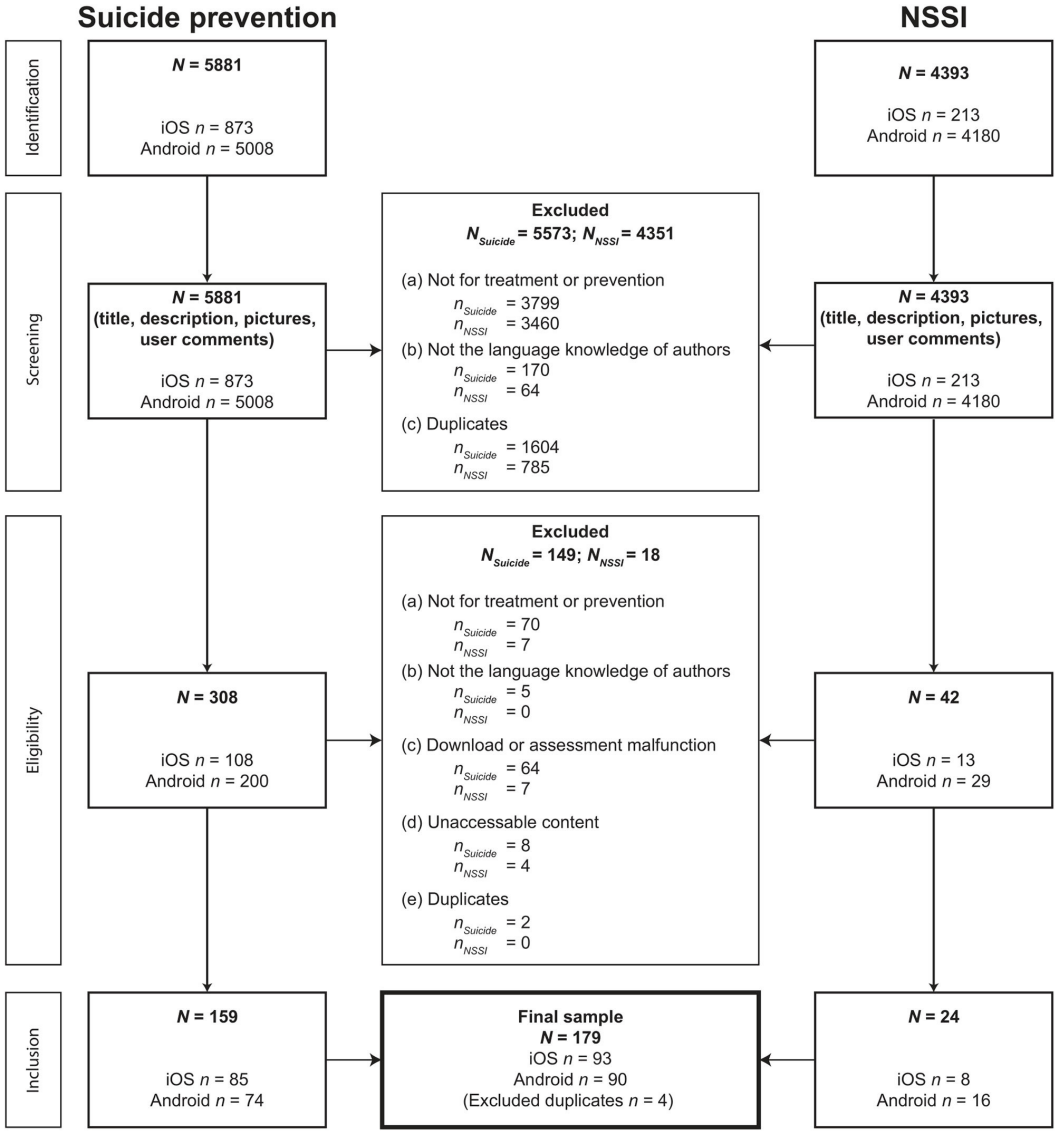
Flowchart of the app inclusion process.

Mobile health applications aimed at preventing suicidal behavior or NSSI were sought, screened, and selected through two distinct, but analogous processes (Figure 1). A total of 10,274 apps were identified by the search engine for suicidal behavior or NSSI. Finally, 183 (1.8%) MHA were included in the analyses; 93 (50.8%) MHA were developed for iOS, and 90 (49.2%) MHA were available for Android. A total of 43 (23.5%) MHA were available for both operating systems. Because four MHA were identified in both searches (suicidality and NSSI), the final sample included *N* = 179 MHA. Some excluded MHA contained triggering content like virtual gun shooting or cliff jumping.

### General Characteristics

Detailed general characteristics of the included MHA are shown in Table 1. Most MHA were developed by commercial companies (*n* = 60; 33.5%) followed by NGOs (*n* = 48; 26.8%), governments (*n* = 43; 24.0%), and universities (*n* = 10; 5.6%). The affiliation of 18 (10.1%) MHA was unknown.

**Table 1:** General characteristics of the included MHA for suicide/NSSI prevention.

	***N***	**(%)**
**Platform**		
iOS Android Both operating systems	92 87 42	51.4 48.6 23.5
**Affiliation**		
Commercial companies Government NGO University Unknown	60 43 48 10 18	33.5 24.0 26.8 5.6 10.1
**Obligatory payment**		
Basic version Extended version/in app purchases	2 5	1.1 2.8
**Involvement in therapy**		
Stand-alone Communication with therapist Sharing of content with the therapist Module assignment by the therapist	149 4 9 8	83.2 2.2 5.0 4.5
**Security and privacy**		
Password Login Privacy policy Informed consent Passive declaration of consent Contact or imprint Secure data transmission	12 5 123 49 84 163 34	6.7 2.8 68.7 27.4 46.9 91.1 19.0
**User rating**		
Apple app Google play	3 48	1.7 26.8
**Technical features**		
Emergency contact Interaction with others In-app community	157 34 4	87.7 19.0 2.2

*MHA, mobile health application; NSSI, non-suicidal self-injury*.

While the basic version of most MHA (*n* = 177; 98.9%) was available for free, payment was mandatory for the basic version of two (1.1%) MHA, the costs were 1.09 € and 7.99 €. Five MHA contained the possibility to buy an upgrade to an extended version or provided in-app purchases, with costs ranging from 0.91 € to 7.99 €.

The majority of MHA (*n* = 149; 83.2%): MHA were stand-alone apps. Only 13 (7.2%) MHA allowed communication (*n* = 4; 2.2%) or sharing of content (*n* = 9; 5.0%) with the therapist. The sharing of content was mainly realized *via* an export function to the local device, which could then be forwarded in a second step. Additionally, only in eight (4.5%) MHA, the module assignment by the therapist was possible.

Password protection was given in 12 (6.7%) MHA, and five (2.8%) MHA required a login. Most MHA provided contact information or legal notice (*n* = 163; 91.1%) and a privacy policy (*n* = 123; 68.7%). About 49 (27%) MHA required active confirmation of informed consent, whereas 84 (46.9%) MHA included a note that consent is automatically given when the app is used. A secure data transmission was provided by 34 (19.0%) MHA. Merely *three* (1.7%) MHA had a user rating in the Apple App store, and 48 (26.8%) MHA in the Google Play store. The mean user rating was 3.96 (SD = 0.72).

A total of 157 (87.7%) MHA offered emergency functions, e.g., helpline numbers or contact information for psychological or medical assistance to be used quickly in case of an acute (suicidal) crisis. In 34 (19.0%) MHA, interaction with others (e.g., *via* Facebook or messenger services) was possible; only four (2.2%) MHA contained in-app communities.

### MHA Quality Rating

Supplementary Table 4 (Appendix C) displays the results of the MARS rating for all subscales of the 179 MHA included. The ICC of the total mean score, indicating agreement of the two reviewers, showed an excellent interclass correlation coefficient (ICC) for both the rating scores of the MHA for suicide prevention (two-way mixed ICC = 0.93; 95% CI: 0.8–0.97) and for the rating scores of the MHA for NSSI (two-way-mixed ICC = 0.90; 95% CI: 0.83–0.96).

The overall quality rating score of MHA for the prevention of suicidal behavior and NSSI was moderate with M = 3.56 (SD = 0.39), ranging from a minimum of 1.88 to a maximum of 4.59. Regarding the four objective subscales, functionality was rated highest (M = 4.14; SD = 0.34; range 2.50–5.0), followed by information quality (M = 3.47; SD = 4.10; range 1.50–4.35), esthetics (M = 3.42; SD = 0.52; range 1.0–5.0), and engagement (M = 3.19; SD = 0.64; range 1.0–4.9). The additional subscales showed lower rating scores: therapeutic gain (M = 2.74; SD = 0.32; range 2.0–4.0), subjective quality (M = 2.72; SD = 0.50; range 1.38–4.0), and perceived impact (M = 2.99; SD = 0.45; range 0.67–4.42).

Fifty-one of the included apps had a user star rating from a minimum of three users. No significant bivariate correlations between the overall total score and the user star ratings were found (*r*[49] = 0.268, *p* > 0.05).

### Evidence

The literature searches revealed published articles on two of the included MHA (“BackUp” and “MYPLAN—your safety plan”). We found no randomized controlled trial on the effectiveness of any included MHA. For MYPLAN, we identified the study protocol of an ongoing randomized controlled trial (39). In addition, there is a qualitative analysis of focus groups with stakeholders (i.e., young vs. adult users of the app, relatives, and clinicians), and qualitative reports of the user involvement in the entire development process (40, 41). BackUp was developed by the Flemish Centre of Expertise in Suicide Prevention (VLESP). A descriptive study tested the usability in an expert panel and an end user panel (42). In addition, we identified a study protocol for a single-arm trial testing the feasibility of a Dutch version of BackUp combined with a self-monitoring app (43).

### Features, Functions, and Suicide/NSSI Prevention Strategies of High-Quality MHA

#### Features and Functions

About 46 (25.14%) MHA with the highest MARS overall rating score (hereinafter referred to as “high-quality MHA”) were examined in more detail for their specific characteristics, functions, and suicide/NSSI prevention strategies. The overall MARS rating score of high-quality MHA was above average with M = 3.91 (SD = 0.24; range 4.53–3.66*). Thirty-one* (67.4%) high-quality MHA were found for iOS and 31 (67.4%) for Android. About 16 (23.9%) high-quality MHA were available for both operating systems. All of the high-quality MHA were free of charge, except for one (2.2%); the basic version of “Gaia Teen Mind” cost 7.99 €. Table 2 displays the features and functions of the high-quality MHA.

**Table 2:** Mean rating scores, features and functions of high-quality MHA for suicide/NSSI prevention.

**Name**	**Subject**	**Platform**	**MARS mean score**	**Target group**	**Security and privacy**	**Evidence and certification**	**Credible source**
BackUp	Suicidal behavior	iOS	4.53	AP AE	PS, CI	WAT Label	✓
Calm harm—manages self-harm	NSSI	iOS/Android	4.43	AP	PW, PS, CI	Award winning	✓
Better stop suicide	Suicidal behavior	Android	4.4	AP	PS, CI	Award winning	✓
AuxiliaApp	Suicidal behavior	iOS/Android	4.32	AP AE P	PW, PS, CI	Web/Aplicación de Psiquiatría-Psicología Acreditada	✓
Krisen Kompass	Suicidal behaviour	iOS/Android	4.31	AP AE	PS, CI		✓
Friend2Friend	Suicidal behavior	iOS	4.26	AE	PS,		✓
distrAct^*^	Suicidal behavior/ NSSI	iOS/Android	4.24	AP	PS, CI	Certified member of the information standard	✓
mhGAP-IG 2.0 App (e-mhGAP)	Suicidal behavior	iOS	4.2	P	PS, CI		✓
Be Safe	Suicidal behavior	iOS	4.19	AP	PS, CI		✓
DMHS: Interactive Suicide Prevention	Suicidal behavior	iOS	4.16	AP	CI		✓
Vrag Maar	Suicidal behavior	iOS/Android	4.14	AP AE	PS, CI		✓
Stay Alive	Suicidal behavior	iOS/Android	4.11	AP AE	PS, CI	Award winning	✓
Operation Life	Suicidal behavior	iOS/Android	4.06	AP	PS, CI		✓
Suicide Prevention App	Suicidal behavior	iOS/Android	4.06	AP AE P	PS, CI		✓
Jewish Care	Suicidal behavior	Android	3.92	AP	PS, CI		✓
Prevent Suicide	Suicidal behavior	iOS	3.89	AP AE	PS, CI	Award winning	✓
Kokua Life	Suicidal behavior	iOS	3.87	AP AE	CI		✓
MoodTools—Depression Aid	Suicidal behavior	iOS/Android	3.87	AP	PS, CI		✓
ReMinder Suicide Safety Plan	Suicidal behavior	iOS/Android	3.87	AP	PS, CI		✓
TechSafe—Mental Health	NSSI	iOS/Android	3.86	AP AE	PS, CI		✓

*AP, affected persons; AE, affiliated environment; P, professionals; PS, privacy policy; CI, contact information; MARS, mobile application rating scale; MHA, mobile health application; NSSI, non-suicidal self-injury; PW, password protection;*

* *after the calculation of the mean score from suicide and NSSI rating; for full version, see Appendix D*.

About 37 (80.4%) high-quality MHA were found with search terms for suicidal behavior and nine with search terms for NSSI. Nonetheless, some high-quality MHA also featured contents for the respective other condition to a notable extent (*n* = 4; 8.7%). On the other hand, three (6.5%) high-quality MHA did not mention either suicidal behavior or NSSI explicitly and did not focus on any specific pathologies, but rather on a general urge management approach, regardless of the associated pathology. A total of 28 (60.9%) high-quality MHA were specifically developed for suicidal behavior, but only two for NSSI (4.3%). Seven (15.2%) high-quality MHA contained a suicide-specific section, eight (17.4%) contained an NSSI-specific section, and four (8.7%) contained a section for both.

Three target groups were addressed by the high-quality MHA, i.e., persons affected by suicidal behavior or NSSI (*n* = 42; 91.3%), their affiliated social environment (*n* = 23; 50.0%), and healthcare professionals (*n* = 6; 13.0%). In addition, some high-quality MHA were aimed at a more specific target group, e.g., the Jewish community (“Jewish Care”), members of the ambulance service in Scotland (“Backup Buddy”), or college students (“Cleveland State Univ Reach Out” and “Lakeland Reach Out”).

The major part of high-quality MHA contained both privacy statement (*n* = 41; 89.1%) and contact information/imprint (*n* = 40; 87.0%). Three (6.5%) MHA also had password protection. The majority of MHA (*n* = 32; 69.6%) could be attributed to a credible source.

A quality labeling (e.g., “certified member of the information standard,” an NHS England quality standard that ensures that the information provided is of high quality and based on the best practice) was found for four (8.7%) MHA. In addition, MHA (*n* = 4; 8.7%) won one or more app-awards, e.g., for their design or user involvement.

#### Purpose and Suicide/NSSI Prevention Strategies

Supplementary Table 6 (Appendix E) displays the purpose, functions, and suicide/NSSI prevention strategies of the 46 high-quality MHA.

Regarding their primary purpose high-quality MHA could be divided into three main categories, i.e, providing information about suicidal behavior/NSSI or general information about mental health (*n* = 36; 78.3%), providing (emergency) resources (*n* = 40; 87.0%), or providing management/reduction of (acute) general or suicidal/NSSI urge and behavior (*n* = 30; 65.2%). In addition, two (4.3%) high-quality MHA provided an assessment tool for the screening of suicidal warning signs/behavior, and one (2.2%) focused on the connection with the therapist.

All of the high-quality MHA included at least three suicide/NSSI prevention strategies. No high-quality MHA included all prevention strategies, but four high-quality MHA were only missing two strategies, whereas 16 (34.8%) high-quality MHA featured self-screening tools to detect suicidal/NSSI risk, and only two (4.3%) included (professional) physician screening tools. The majority high-quality MHA encouraged or facilitated access to peer or family support functions (*n* = 41; 89.1%), 32 (69.6%) to non-crisis support functions, and 37 (80.4%) to crisis support/emergency hotlines. In 18 (39.1%) high-quality MHA, the crisis support/emergency hotline was visible at all times within the MHA.

The high-quality MHA represented a number of mental health strategies focused on preventing suicide/NSSI. All of the high-quality MHA delivered/provided some kind of psychotherapy, some more extensively than others. The psychotherapeutic contents that were provided were: tips/advice (*n* = 41; 89.1%), information/education (*n* = 36; 78.3%), strategies/skills (*n* = 31; 67.4%), and urge management (*n* = 29; 63.0%). Additionally, one high-quality MHA also provided mood tracking and problem-solving content.

An individual safety plan could be created in 25 (54.3%) high-quality MHA, and 12 (26.1%) high-quality MHA gave information on restricting access to lethal means (e.g., firearms and drugs).

Out of all high-quality MHA, the majority (*n* = 30 (65.2%) included sections to identify individual warning signs in the MHA user (e.g., as part of a personalized safety plan) or general warning signs in others. Furthermore, 17 (37.0%) high-quality MHA allowed users to identify personal triggers.

All of the high-quality MHA, except one (2.2%), either allowed users to enter their own personalized coping strategies and/or provided a selection of predefined coping strategies for users to choose from. The coping strategies used were: resource orientation (*n* = 43; 93.5%), distraction (*n* = 25, 54.3%), positive reminders (*n* = 15; 32.6%), relaxation (*n* = 13; 28.3%), mindfulness (*n* = 11; 24.0%), breathing (*n* = 10; 21.7%), comfort (*n* = 10; 21.7%), grounding (*n* = 9; 19.6%), exercise (*n* = 8; 17.4%), self-expression (*n* = 6; 13.0%), acceptance (*n* = 5; 10.9%), and mental training (*n* = 2; 4.3%).

## Discussion

### Key Findings

This is the first study systematically examining publicly available MHA for the prevention of suicidal behavior and NSSI in European commercial app stores (Apple App Store and Google Play). The search engine revealed a large number of available apps (*n* = 10,274), of which 183 (1.8%) were operable and included a specific content for the prevention of suicidal behavior or NSSI. The large number of irrelevant apps, in particular due to the Google Play Store, may make it difficult for users and healthcare providers to identify relevant MHA. In addition, user ratings displayed in app store descriptions showed no significant correlation with the overall quality rating scores, which is consistent with previous findings showing that user ratings are not an appropriate indicator to guide users (29, 30, 44–47). Even more alarming is the fact that several excluded apps that were found alienate the terminology of the conditions in a potentially harmful way. This included, for example, games that involve virtual gun shooting or cliff jumping, which can lead to uncontrolled exposure to potential triggers that, according to the theory of “suicide contagion,” can negatively impact symptom severity in persons with suicidal ideation (48).

A further concern relates to the absence of an evidence base of the included MHA. For only two MHA, there are descriptive or qualitative reports on the development process and preliminary feasibility (“MYPLAN” and “BackUp”) (40, 42). Feedback from stakeholders, including participants who were suicidal and clinicians, indicates the potential of these MHA for clinical use. However, only for one MHA (“MYPLAN”; Andreasson et al., (39)), a study protocol for a randomized controlled trial examining the effectiveness of a mobile app-safety plan to reduce suicidal ideation could be found. This finding is consistent with prior investigations of MHA for mental disorders (30, 45, 47, 49) and may lead to potential iatrogenic effects of the MHA (50).

Yet, 183 functioning and relevant MHA with an average moderate MARS quality (M = 3.56) were identified. The MARS subscale rating revealed that the assessed MHA typically functions well, but shows deficiencies in information quality, esthetics, and engagement. The primary focus of 40 high-quality MHA was to provide helpful resources for persons affected with suicide/NSSI ideation or who are in an acute crisis and to provide contact information to crisis support (phone numbers, addresses, or links), medical facilities, or groups or personal contacts (e.g., family and social environment). In addition, a notable number of MHA addressed specific target groups, e.g., veterans or college students, who are known to be at increased risk for suicide (18, 51). Clinicians interested in integrating MHA into their clinical practice should consider the usefulness of the respective app to achieve specific goals in treatment plan of the patient. Databases, such as the MHAD (http://mhad.science) or the APA app advisor (https://www.psychiatry.org/psychiatrists/practice/mental-health-apps), can provide guidance to clinicians.

The suicide prevention tools used in high-quality MHA (Supplementary Table 6) largely reflected established best practice suicide prevention strategies (e.g., facilitating peer or family support). Numerous high-quality MHA included either physician- or self-screening tools for the detection of suicidal risk. All high-quality MHA contained strategies for accessing support through a variety of sources. Contrary to the results of previous analyses in this area, the present study identified a number of MHA that included multiple suicide prevention strategies (20). This suggests a positive trend in the development of MHA for suicide prevention in recent years.

Although the majority of high-quality MHA included valuable crisis- or non-crisis support functions, these resources were often only limited to a specific geographical area. This information should be provided more consistently in the app store description. Furthermore, Martinengo et al. (52) reported erroneous helpline numbers in some suicide prevention MHA, potentially representing another serious risk for users.

Only about half of the high-quality MHA focused on creating a safety plan as a treatment strategy. Those safety plans further differ in their features and comprehensiveness and vary in the degree of adherence to the guidelines proposed by Stanley and Brown (53). Exhaustive standardized safety plans, containing a list of warning signs, coping and socialization strategies, contact information, and information on the restriction of access to means, represent an easily manageable and always available tool in MHA for suicide/NSSI prevention and should be more widely incorporated.

The widespread presence of privacy features in high-quality MHA is positively surprising, given the results of prior MHA evaluations (20, 29, 30, 47). Especially with regard to suicidal behavior, sensitive handling of personal data is essential, as stigmatization is one of the key barriers to professional help-seeking of persons at elevated risk for suicide (18).

Furthermore, it is encouraging that the majority of high-quality MHA (*n* = 32) could be assigned to credible sources such as NGOs, governmental institutions, or universities.

### Limitations

This study has some limitations. First, it is possible that not all relevant MHA were found. Google Play and Apple App Store limit the number of results per search term, and this limitation applies also to the search engine provided on the MHAD platform. Furthermore, some MHA are limited to certain geographic areas (e.g., by the publisher), since this search was limited to the European stores of some relevant MHA, which are only available in non-European regions, were not identifiable. Yet, a large number of the identified MHA were developed for the non-European market (e.g., USA and Canada), but also available in the European stores. This suggests that our search may be representative for the global app market. Moreover, the app market is expanding quickly, and a new search and rating process could lead to different search and rating results (54). This is evidenced by the fact that one high-quality MHA was already unavailable during the course of assessment.

Second, consistent with other studies (45, 47, 55), this study systematically evaluated MHA using the MARS and is an important step toward identifying high-quality MHA. However, the MARS only reliably analyzes app quality in terms of engagement, functionality, aesthetics, information quality, therapeutic gain, subjective quality, and impact factor. A high mean score in the MARS rating neither provides information about the effectiveness of an MHA, nor about its usefulness. Thus, it is unclear whether the high-quality MHA found for suicide/NSSI prevention are actually considered helpful by affected persons, their affiliated environment, or healthcare professionals. Considering that previous studies found deficiencies in the functionality (52) and information quality (20) of some MHA for suicide prevention, a more in-depth analysis of the functions provided and their effectiveness would be useful. Third, the search for MHA for NSSI was only conducted in German and English languages. However, MHA in Dutch identified by this search were included in the evaluation.

### Conclusion

Our investigation showed that there are numerous MHA for suicide/NSSI prevention available in the European commercial app stores, some of high quality, incorporating evidence-based suicide prevention measures and thus potentially able to support affected persons, their affiliated environment, or healthcare professionals. Given the limited interpersonal contact opportunities due to the measures to contain the COVID-19 pandemic, high-quality MHA for the prevention of suicidal behavior and NSSI can be a necessary and valuable source of assistance for affected persons. However, because of an absent evidence base on effectiveness, the benefits and harms of the included MHA cannot be evaluated. Furthermore, users may have great difficulty finding an appropriate MHA with suitable content, due to a plethora of irrelevant apps including apps with potential harmful content. These results suggest that the current free availability of MHA for mental disorders may need to be more regulated to protect users. The results of this review will be made publicly available on the MHAD (http://mhad.science) to guide users and clinicians and provide a greater transparency regarding MHA quality. To unfold their full potential, high-quality MHA containing multifaceted suicide prevention strategies should be made available in every county and in every language.

## Data Availability Statement

The raw data supporting the conclusions of this article will be made available by the authors, without undue reservation.

## Author Contributions

LS, YT, and E-MM initiated this study. LS, YT, E-MM, M-LL, RV, and HB contributed to the study design. LS, M-LL, and RV drafted the manuscript and ran the analysis. M-LL, RV, and EDJ rated MHA. LS supervised the ratings. RB performed the literature review. All authors revised the manuscript and approved the final version.

## Conflict of Interest

LS, E-MM, HB, and YT developed and run the German Mobile Health App Database (MHAD) project. The MHAD is a self-funded project at Ulm University without commercial interests. HB, LS, and E-MM received payments for talks and workshops in the context of e-mental-health. The remaining authors declare that the research was conducted in the absence of any commercial or financial relationships that could be construed as a potential conflict of interest.

## Publisher's Note

All claims expressed in this article are solely those of the authors and do not necessarily represent those of their affiliated organizations, or those of the publisher, the editors and the reviewers. Any product that may be evaluated in this article, or claim that may be made by its manufacturer, is not guaranteed or endorsed by the publisher.
