# Supplementary material for: A Systematic Evaluation of Mobile Health Applications for the Prevention of Suicidal Behavior or Non-suicidal Self-injury
Source: Front Digit Health. 2021 Jul 26;3:689692. doi: 10.3389/fdgth.2021.689692 (PMC8521855; doi:10.3389/fdgth.2021.689692)
Supplement: Supplementary file 1 [file Data_Sheet_1.pdf]

Supplementary Material

1 Supplementary Tables

1.1 Appendix A: Full Lists of Search Terms (App Store)

Supplementary Table 1. Full list of search terms for suicidal behaviour

English	Spanish	Dutch	German
suicide	suicidio	zelfmoord	Suizid
suicide prevention	prevención del suicidio	zelfmoordpreventie	Suizidprävention
suicidal ideation	ideación suicida	zelfmoordgedachten	Suizidgedanken
suicide attempt	intento de suicidio	zelfmoordpoging	Suizidversuch
suicide risk	riesgo de suicidio	zelfmoordrisico	Suizidrisiko
suicidal thoughts	pensamientos suicidas	zelfmoordgedachten	Suizidgedanken
suicidal behavio[u]r	comportamiento suicida	suïcidaal gedrag	Suizidverhalten
suicidality	suicidio	zelfmoord	Suizidalität

Supplementary Table 2. Full list of search terms NSSI

English	German
self-injury	
selfinjury	Selbstverletzung
self injury	
self-harm	Selbstschädigung
selfharm	Selbstbeschädigung
self harm	
self-mutilation	
selfmutilation	
self mutilation	
auto-mutilation	
automutilation	
self-injurious behavior	Selbstverletzendes Verhalten
selfinjurious behavior	

self injurious behavior

Self-destruction

self destruction

parasuicide

para suicide

1.2 Appendix B: Full List of Search Terms (Database Search)

Supplementary Table 3. Full list of search terms for evidence on identified MHA

Pubmed	PsycINFO via Ebsco
(BackUp OR	(BackUp OR
Calm Harm OR	Calm Harm OR
Calm Harm - manages self harm OR	Calm Harm - manages self harm OR
Better Stop Suicide OR	Better Stop Suicide OR
AuxiliaApp OR	AuxiliaApp OR
AuxiliaApp OR	AuxiliaApp OR
Krisen Kompass OR	Krisen Kompass OR
Krisen Kompass OR	Krisen Kompass OR
distrACT OR	distrACT OR
distrACT OR	distrACT OR
Friend2Friend OR "Friend 2 Friend"	Friend2Friend OR "Friend 2 Friend"
distrACT OR	distrACT OR
distrACT OR	distrACT OR
mhGAP-IG 2.0 App (e-mhGAP) OR mhGAP-IG 2.0 App OR	mhGAP-IG 2.0 App (e-mhGAP) OR mhGAP-IG 2.0 App OR
Be Safe OR	Be Safe OR
DMHS: Interactive Suicide Prevention OR Interactive Suicide Prevention OR	DMHS: Interactive Suicide Prevention OR Interactive Suicide Prevention OR
Vrag Maar OR	Vrag Maar OR
Vrag Maar OR	Vrag Maar OR
Stay Alive OR	Stay Alive OR
Stay Alive OR	Stay Alive OR
Suicide Prevention App OR	Suicide Prevention App OR
Operation Life OR	Operation Life OR
Operation Life OR	Operation Life OR
Suicide Prevention App OR	Suicide Prevention App OR
ReMinder Suicide Safety Plan OR	ReMinder Suicide Safety Plan OR
Jewish Care OR	Jewish Care OR
Prevent Suicide OR	Prevent Suicide OR
Kokua Life OR	Kokua Life OR
MoodTools - Depression Aid OR MoodTools OR	MoodTools - Depression Aid OR MoodTools OR
MoodTools - Depression Aid OR	MoodTools - Depression Aid OR
TechSafe - Mental Health OR	TechSafe - Mental Health OR
TechSafe - Mental Health OR	TechSafe - Mental Health OR
OnTrackAgain OR	OnTrackAgain OR
OnTrackAgain OR	OnTrackAgain OR
Prevent Suicide - D&G OR Prevent suicide OR	Prevent Suicide - D&G OR Prevent suicide OR
Prevent Suicide - Highland OR	Prevent Suicide - Highland OR
Prevent Suicide - Highland OR	Prevent Suicide - Highland OR
Prevent Suicide - NE Scotland OR	Prevent Suicide - NE Scotland OR
Prevensuic OR	Prevensuic OR
Prevensuic OR	Prevensuic OR
Elevate Client OR	Elevate Client OR
Christian Veterinary Mission OR	Christian Veterinary Mission OR
Suïcide Preventie AppOR suicide preventie app OR	Suïcide Preventie AppOR suicide preventie app OR
Suïcide Preventie AppOR	Suïcide Preventie AppOR
Cleveland State Univ Reach Out OR Cleveland state university reach out OR	Cleveland State Univ Reach Out OR Cleveland state university reach out OR
Suicide Safety Plan OR	Suicide Safety Plan OR
The Daily Difference OR	The Daily Difference OR
I live OR	I live OR
Brothers for Life OR	Brothers for Life OR
Student Health App OR	Student Health App OR
ReMinder Suicide Safety Plan OR	ReMinder Suicide Safety Plan OR
Suicide Safety Plan OR	Suicide Safety Plan OR
DMHS: Suicide Prevention Info OR	DMHS: Suicide Prevention Info OR
Carpe Diem - Depression and Anxiety Forum OR	Carpe Diem - Depression and Anxiety Forum OR
SafetyNet: Your Suicide Prevention App OR safety net OR	SafetyNet: Your Suicide Prevention App OR safety net OR
Columbus State Hope & Help OR	Columbus State Hope & Help OR
MYPLAN - Your safety plan OR MYPLAN OR	MYPLAN - Your safety plan OR MYPLAN OR
Lakeland Reach Out OR	Lakeland Reach Out OR
Students Against Violence MS OR	Students Against Violence MS OR
Backup Buddy (SCOT) OR	Backup Buddy (SCOT) OR

DMHS Suicide Prevention and Crisis Access Linkage Line (C.A.L.L.) OR C.A.L.L. OR
Tri-C Help Is Here OR
Backup Buddy (SECAMB) OR
Gaia Teen Mind OR
ReliefLink OR
Blue Life Coach OR
MY3 - Support Network OR
MY3 - Support Network OR
Reach Out-Univ of Cincinnati OR Reach out OR
Reach Out-Case Western Reserve OR
Mount Union Raiders Reach Out OR
HOPE - Broome County Mental Health Department OR
Calm Care OR
Calm Care OR
Sinclair College Hope Link OR
Onondaga SPS OR
Onondaga SPS OR
Columbia Protocol OR
Columbia Protocol OR
Dutchess County HELPLINE OR
iHelp Sunshine Coast OR
Combined Minds OR
WeCare, Aviation OR wecare OR
TheHopeLine OR
Self-Heal OR
WeCare, 7th SFG(A) OR
WeCare, 1st Theater Sustainment Command OR
WeCare, 428th FA BDE OR
WeCare, 7th TB(X) OR
WeCare, Fort Stewart OR
WeCare, Walter Reed OR
Albany County HOPE OR
Ulster County SPEAK OR
The Fire Watch OR
WeCare Fort Huachuca OR
WeCare JBLE OR
WeCare JBM-HH OR
WeCare, Camp Humphreys OR
WeCare, Europe OR
WeCare, Fort Campbell OR
WeCare, Fort Campbell OR
WeCare, Fort Detrick OR
WeCare, Fort Detrick OR
WeCare, Fort Hood OR
WeCare, Fort Jackson OR
WeCare, Fort Rucker OR
WeCare, Ft Bragg OR
WeCare, GA NG OR
WeCare, GA NG OR
WeCare, JBSA OR
WeCare, JBSA OR
WeCare, JRTC & Fort Polk OR
WeCare, Resolute Support OR
WeCare, USACE OR
WeCare, USACE OR
WeCare, USAJFKSWCS OR
WeCare, USAJFKSWCS OR
WeCare, USAREC OR
WeCare, 7th TB (X) OR
Coaches Assistance Program OR
Self-Heal OR
Don't panic - Depression and panic help OR don't panic OR
Suicide? Help! Tayside OR suicide help OR
trustTalk247 OR
Ulster County SPEAK OR
STOPP app OR
oscER Jr. San Diego OR
oscER San Diego OR
WeCare, CASCOM OR
SPEAK North Alabama OR
Albany County HOPE OR
Elijah OR
SPEAK North Alabama OR
Urgencias psiquiátricas OR
WeCare CASCOM OR
iwontbesilent OR i won't be silent OR

DMHS Suicide Prevention and Crisis Access Linkage Line (C.A.L.L.) OR C.A.L.L. OR
Tri-C Help Is Here OR
Backup Buddy (SECAMB) OR
Gaia Teen Mind OR
ReliefLink OR
Blue Life Coach OR
MY3 - Support Network OR
MY3 - Support Network OR
Reach Out-Univ of Cincinnati OR Reach out OR
Reach Out-Case Western Reserve OR
Mount Union Raiders Reach Out OR
HOPE - Broome County Mental Health Department OR
Calm Care OR
Calm Care OR
Sinclair College Hope Link OR
Onondaga SPS OR
Onondaga SPS OR
Columbia Protocol OR
Columbia Protocol OR
Dutchess County HELPLINE OR
iHelp Sunshine Coast OR
Combined Minds OR
WeCare, Aviation OR wecare OR
TheHopeLine OR
Self-Heal OR
WeCare, 7th SFG(A) OR
WeCare, 1st Theater Sustainment Command OR
WeCare, 428th FA BDE OR
WeCare, 7th TB(X) OR
WeCare, Fort Stewart OR
WeCare, Walter Reed OR
Albany County HOPE OR
Ulster County SPEAK OR
The Fire Watch OR
WeCare Fort Huachuca OR
WeCare JBLE OR
WeCare JBM-HH OR
WeCare, Camp Humphreys OR
WeCare, Europe OR
WeCare, Fort Campbell OR
WeCare, Fort Campbell OR
WeCare, Fort Detrick OR
WeCare, Fort Detrick OR
WeCare, Fort Hood OR
WeCare, Fort Jackson OR
WeCare, Fort Rucker OR
WeCare, Ft Bragg OR
WeCare, GA NG OR
WeCare, GA NG OR
WeCare, JBSA OR
WeCare, JBSA OR
WeCare, JRTC & Fort Polk OR
WeCare, Resolute Support OR
WeCare, USACE OR
WeCare, USACE OR
WeCare, USAJFKSWCS OR
WeCare, USAJFKSWCS OR
WeCare, USAREC OR
WeCare, 7th TB (X) OR
Coaches Assistance Program OR
Self-Heal OR
Don't panic - Depression and panic help OR don't panic OR
Suicide? Help! Tayside OR suicide help OR
trustTalk247 OR
Ulster County SPEAK OR
STOPP app OR
oscER Jr. San Diego OR
oscER San Diego OR
WeCare, CASCOM OR
SPEAK North Alabama OR
Albany County HOPE OR
Elijah OR
SPEAK North Alabama OR
Urgencias psiquiátricas OR
WeCare CASCOM OR
iwontbesilent OR i won't be silent OR

Be Safe OR	Be Safe OR
WeCare, JRTC & Fort Polk OR	WeCare, JRTC & Fort Polk OR
Jason Foundation A Friend Ask OR	Jason Foundation A Friend Ask OR
A.L.E.R.T.OR	A.L.E.R.T.OR
Am I? Safety Plan OR	Am I? Safety Plan OR
Operation Reach Out OR	Operation Reach Out OR
Jason Foundation A Friend Ask OR	Jason Foundation A Friend Ask OR
Coping Skills OR	Coping Skills OR
Step Up and Speak Out OR	Step Up and Speak Out OR
Step Up and Speak Out OR	Step Up and Speak Out OR
Guard Your Buddy - Tennessee OR guard your buddy OR	Guard Your Buddy - Tennessee OR guard your buddy OR
Be Safe OR	Be Safe OR
HELP App-Prevention ResourcesOR	HELP App-Prevention ResourcesOR
WeCare 59th Ordnance Brigade OR	WeCare 59th Ordnance Brigade OR
WeCare, 59th Ordnance BDE OR	WeCare, 59th Ordnance BDE OR
Shatter the Silence OR	Shatter the Silence OR
MS DMH - Shatter the Silence OR	MS DMH - Shatter the Silence OR
Hywel Dda Self Help Guides OR	Hywel Dda Self Help Guides OR
Operation Reach Out OR	Operation Reach Out OR
The LifeLine OR	The LifeLine OR
The LifeLine OR	The LifeLine OR
CerboCare : OCD and Depression Relief Self Help OR	CerboCare : OCD and Depression Relief Self Help OR
Community Stress First Aid OR	Community Stress First Aid OR
Alachua Talk OR	Alachua Talk OR
MSE&SUICIDE ASSESSr OR	MSE&SUICIDE ASSESSr OR
Is Someone Suicidal? OR	Is Someone Suicidal? OR
TUFMINDS OR	TUFMINDS OR
eDIVO OR	eDIVO OR
Suicide Lifeguard OR	Suicide Lifeguard OR
Don't panic - Depression and panic help OR	Don't panic - Depression and panic help OR
HELP Prevent Suicide OR	HELP Prevent Suicide OR
HELP Prevent Suicide OR	HELP Prevent Suicide OR
Emotional Support Helpline Directory OR	Emotional Support Helpline Directory OR
Prevención del suicidio OR	Prevención del suicidio OR
Self Harm Recovery OR	Self Harm Recovery OR
First Step Oregon OR	First Step Oregon OR
Suicidio y Psicología OR	Suicidio y Psicología OR
Self Harm Recovery OR	Self Harm Recovery OR
TheHopeLine OR	TheHopeLine OR
Suicide Prevention -Ways to Help a Suicidal Friend OR	Suicide Prevention -Ways to Help a Suicidal Friend OR
PMCS Combating Suicide OR combating suicide OR	PMCS Combating Suicide OR combating suicide OR
Safe Hearts OR	Safe Hearts OR
A Teen Suicide Prevention Anime OR	A Teen Suicide Prevention Anime OR
Alaska Careline OR	Alaska Careline OR
Army Leader Smart Cards OR	Army Leader Smart Cards OR
Mental Health - psychologist OR	Mental Health - psychologist OR
Botón Anti-Suicidio OR	Botón Anti-Suicidio OR
BackUp door Zelfmoord OR	BackUp door Zelfmoord OR
suicid* OR self-harm OR selfharm OR "self harm")	suicid* OR self-harm OR selfharm OR "self harm")
AND	AND
(app[Title/Abstract] OR mobile[Title/Abstract])	(TI (app OR mobile) OR AB (app OR mobile))

1.3 Appendix C: MARS Ratings

Supplementary Table 4. Mean MARS rating scores in descending order of the total mean score

	Platform	Search Terms	Overall mean score	Engagement	Functionality	Esthetics	Information Quality	Therapeutic Gain	Subjective Quality	Impact Factor
BackUp	iOS	Suicidality	4,59	4,60	5,00	5,00	3,75	4,00	3,75	3,67
Calm Harm - manages self harm	Android	NSSI	4,43	4,50	4,50	4,83	3,90	3,50	4,00	3,75
Calm Harm - manages self harm	iOS	NSSI	4,43	4,40	4,50	4,83	3,98	3,50	3,88	3,75
Better Stop Suicide	Android	Suicidality	4,40	4,90	4,38	4,33	4,00	3,33	3,75	3,83
AuxiliaApp	Android	Suicidality	4,32	4,20	4,75	4,33	4,00	3,00	3,75	3,67
AuxiliaApp	iOS	Suicidality	4,32	4,20	4,75	4,33	4,00	3,00	3,80	3,67
Krisen Kompass	iOS	Suicidality	4,31	4,40	4,38	4,17	4,29	3,42	3,75	3,92
Krisen Kompass	Android	Suicidality	4,31	4,40	4,38	4,17	4,29	3,42	3,75	3,92
distrACT	iOS	Suicidality/NSSI	4,24	3,70	4,69	4,42	4,15	3,17	3,63	4,04
distrACT	Android	Suicidality/NSSI	4,24	3,70	4,69	4,42	4,15	3,17	3,63	4,04
Friend2Friend	iOS	Suicidality	4,26	4,50	4,38	4,00	4,17	3,00	3,75	3,83
mhGAP-IG 2.0 App (e-mhGAP)	iOS	Suicidality	4,20	3,80	4,63	4,17	4,20	3,25	3,75	2,59
Be Safe	iOS	Suicidality	4,19	4,00	4,75	4,17	3,84	2,84	3,50	3,50
DMHS: Interactive Suicide Prevention	iOS	Suicidality	4,16	4,60	4,25	4,00	3,80	3,17	3,75	3,67
Vrag Maar	Android	Suicidality	4,14	4,20	4,75	4,00	3,60	3,00	3,00	3,50
Vrag Maar	iOS	Suicidality	4,14	4,20	4,75	4,00	3,60	3,00	3,00	3,50
Stay Alive	iOS	Suicidality	4,11	4,40	4,38	4,00	3,68	3,67	3,75	3,50
Stay Alive	Android	Suicidality	4,11	4,40	4,38	4,00	3,67	3,67	3,75	3,50
Suicide Prevention App	Android	Suicidality	4,08	4,00	4,50	4,00	3,83	3,00	3,50	3,33
Operation Life	Android	Suicidality	4,06	4,20	4,25	4,00	3,80	3,13	3,38	3,25
Operation Life	iOS	Suicidality	4,06	4,20	4,25	4,00	3,80	3,13	3,63	3,25
Suicide Prevention App	iOS	Suicidality	4,03	3,90	4,38	4,00	3,83	3,00	3,38	3,25
ReMinder Suicide Safety Plan	iOS	Suicidality	4,00	4,20	4,25	4,00	3,53	3,00	3,50	3,17
Jewish Care	Android	Suicidality	3,92	4,20	4,25	3,83	3,40	3,00	2,75	3,50
Prevent Suicide	iOS	Suicidality	3,89	3,90	4,25	4,00	3,40	3,00	3,25	3,34
Kokua Life	iOS	Suicidality	3,87	3,80	3,88	4,00	3,80	2,88	2,75	3,34
MoodTools - Depression Aid	Android	Suicidality	3,87	3,80	4,25	3,83	3,59	3,17	3,38	3,08
MoodTools - Depression Aid	iOS	Suicidality	3,87	3,80	4,25	3,83	3,59	3,17	3,38	3,08
TechSafe - Mental Health	iOS	NSSI	3,86	2,80	4,50	4,17	3,98	3,00	2,75	3,58
TechSafe - Mental Health	Android	NSSI	3,86	2,80	4,50	4,17	3,98	3,00	2,88	3,58
OnTrackAgain	Android	Suicidality	3,83	3,80	4,00	4,00	3,50	3,00	3,25	3,17
OnTrackAgain	iOS	Suicidality	3,83	3,80	4,00	4,00	3,50	3,00	3,25	3,17
Prevent Suicide - D&G	iOS	Suicidality	3,82	3,70	4,25	3,84	3,50	3,00	3,00	3,34
Prevent Suicide - Highland	Android	Suicidality	3,82	3,70	4,25	3,84	3,50	3,00	3,00	3,34
Prevent Suicide - Highland	iOS	Suicidality	3,82	3,70	4,25	3,84	3,50	3,00	3,00	3,34
Prevent Suicide - NE Scotland	Android	Suicidality	3,81	3,70	4,25	3,84	3,47	3,00	3,00	3,34

Prevensuic	Android	Suicidality	3,81	3,80	4,25	3,67	3,50	3,00	2,75	3,00
Prevensuic	iOS	Suicidality	3,81	3,80	4,25	3,67	3,50	3,00	2,75	3,00
Elevate Client	Android	Suicidality	3,80	4,00	3,88	3,67	3,67	3,25	2,75	3,25
Christian Veterinary Mission	Android	Suicidality	3,79	3,20	4,25	4,50	3,20	2,17	2,63	2,25
Suïcide Preventie App	Android	Suicidality	3,79	3,80	4,25	3,70	3,40	2,75	3,00	2,50
Suïcide Preventie App	iOS	Suicidality	3,79	3,80	4,25	3,70	3,40	2,75	3,00	2,50
Cleveland State Univ Reach Out	iOS	Suicidality	3,78	2,90	4,63	3,50	4,10	2,50	2,88	3,33
Suicide Safety Plan	iOS	Suicidality	3,78	3,80	4,25	3,67	3,40	2,71	3,00	3,00
The Daily Difference	Android	NSSI	3,78	3,00	4,38	3,83	3,90	3,00	3,38	3,67
I live	Android	Suicidality	3,77	4,00	4,13	3,67	3,30	2,84	2,75	3,33
Brothers for Life	iOS	Suicidality	3,75	3,80	4,38	3,67	3,17	2,67	3,13	2,92
Student Health App	Android	NSSI	3,75	3,20	4,13	3,33	4,35	3,00	2,88	3,50
ReMinder Suicide Safety Plan	Android	Suicidality	3,75	4,20	4,25	3,00	3,53	2,67	3,50	3,17
Suicide Safety Plan	Android	Suicidality	3,74	3,80	4,25	3,50	3,40	2,71	3,00	3,00
DMHS: Suicide Prevention Info	Android	Suicidality	3,72	3,30	4,25	3,67	3,67	3,17	2,75	3,00
Carpe Diem - Depression and Anxiety Forum	Android	Suicidality	3,71	4,00	4,50	3,50	2,83	2,33	2,75	2,42
SafetyNet: Your Suicide Prevention App	Android	Suicidality	3,71	3,70	4,13	3,67	3,33	2,83	2,75	2,75
Columbus State Hope & Help	iOS	Suicidality	3,69	2,90	4,38	3,67	3,84	2,67	2,75	3,17
MYPLAN - Your safety plan	Android	Suicidality	3,69	3,60	4,00	3,67	3,50	3,83	3,13	3,00
Lakeland Reach Out	iOS	Suicidality	3,69	2,80	4,38	3,67	3,90	2,50	2,88	3,25
Students Against Violence MS	iOS	NSSI	3,68	2,40	4,50	3,83	4,00	2,83	2,50	2,92
Backup Buddy (SCOT)	Android	NSSI	3,68	2,90	4,13	3,83	3,88	2,67	2,13	3,33
DMHS Suicide Prevention and Crisis Access Linkage Line (C.A.L.L.)	iOS	Suicidality	3,68	3,30	4,25	3,50	3,67	3,17	2,75	3,09
Tri-C Help Is Here	iOS	Suicidality	3,66	3,00	4,25	3,50	3,90	2,67	2,75	3,25
Backup Buddy (SECAMB)	Android	NSSI	3,66	2,80	4,13	3,83	3,88	2,67	2,13	3,33
Gaia Teen Mind	Android	NSSI	3,66	2,80	4,75	3,33	3,75	2,83	2,50	3,33
ReliefLink	iOS	Suicidality	3,65	3,70	4,00	3,50	3,42	3,13	2,75	2,92
Blue Life Coach	iOS	Suicidality	3,65	3,60	3,88	3,33	3,80	3,00	3,38	3,50
MY3 - Support Network	iOS	Suicidality	3,65	3,80	4,00	4,00	2,80	2,67	2,75	2,83
MY3 - Support Network	Android	Suicidality	3,65	3,80	4,00	4,00	2,80	2,67	2,75	2,83
Reach Out-Univ of Cincinnati	iOS	Suicidality	3,64	3,00	4,25	3,50	3,80	2,67	2,75	3,25
Reach Out-Case Western Reserve	iOS	Suicidality	3,64	3,00	4,25	3,50	3,80	2,67	2,88	3,25
Mount Union Raiders Reach Out	iOS	Suicidality	3,62	2,80	4,38	3,50	3,80	2,67	2,50	3,00
HOPE - Broome County Mental Health Department	iOS	Suicidality	3,62	2,90	4,25	3,50	3,82	2,67	2,75	2,75
Calm Care	iOS	Suicidality	3,61	3,20	4,25	3,33	3,67	2,33	2,50	2,75
Calm Care	Android	Suicidality	3,61	3,20	4,25	3,33	3,67	2,33	2,50	2,75
Sinclair College Hope Link	iOS	Suicidality	3,61	3,20	4,25	3,50	3,50	2,67	2,75	3,17
Onondaga SPS	iOS	Suicidality	3,60	3,00	4,38	3,50	3,53	2,67	2,63	3,00
Onondaga SPS	Android	Suicidality	3,60	3,00	4,38	3,50	3,53	2,67	2,63	3,00
Columbia Protocol	Android	Suicidality	3,58	3,40	4,25	3,33	3,33	2,33	2,13	2,75
Columbia Protocol	iOS	Suicidality	3,58	3,40	4,25	3,33	3,33	2,33	2,13	2,75

Dutchess County HELPLINE	iOS	Suicidality	3,57	3,50	3,88	3,33	3,58	2,67	2,63	3,42
iHelp Sunshine Coast	iOS	Suicidality	3,57	2,80	4,25	3,83	3,40	2,50	2,63	2,92
Combined Minds	iOS	NSSI	3,56	2,50	4,13	4,00	3,60	3,00	3,00	3,33
WeCare, Aviation	Android	Suicidality	3,55	3,00	4,25	3,17	3,80	2,67	2,50	3,08
TheHopeLine	Android	Suicidality/NSSI	3,33	3,40	3,69	3,25	2,98	2,50	2,26	2,79
Self-Heal	Android	NSSI	3,55	3,10	3,88	3,33	3,88	3,00	2,88	3,67
WeCare, 7th SFG(A)	Android	Suicidality	3,53	3,00	4,25	3,17	3,70	2,67	2,63	3,00
WeCare, 1st Theater Sustainment Command	iOS	Suicidality	3,53	3,00	4,25	3,17	3,70	2,83	2,63	3,00
WeCare, 428th FA BDE	iOS	Suicidality	3,53	3,00	4,25	3,17	3,70	2,67	2,50	3,08
WeCare, 7th TB(X)	Android	Suicidality	3,53	3,00	4,25	3,17	3,70	2,67	2,50	3,08
WeCare, Fort Stewart	iOS	Suicidality	3,53	3,00	4,25	3,17	3,70	2,67	2,50	3,08
WeCare, Walter Reed	iOS	Suicidality	3,53	3,10	4,25	3,17	3,60	2,67	2,63	3,00
Albany County HOPE	Android	Suicidality	3,52	3,50	3,75	3,33	3,50	2,67	2,75	3,42
Ulster County SPEAK	Android	Suicidality	3,52	3,30	4,25	3,17	3,35	2,67	2,50	2,92
The Fire Watch	iOS	Suicidality	3,51	3,10	4,13	3,50	3,30	2,50	2,50	2,83
WeCare Fort Huachuca	iOS	Suicidality	3,50	3,00	4,25	3,17	3,60	2,67	2,63	3,00
WeCare JBLE	iOS	Suicidality	3,50	3,00	4,25	3,17	3,60	2,67	2,63	3,00
WeCare JBM-HH	iOS	Suicidality	3,50	3,00	4,25	3,17	3,60	2,67	2,63	3,00
WeCare, Camp Humphreys	Android	Suicidality	3,50	3,00	4,25	3,17	3,60	2,67	2,63	3,00
WeCare, Europe	Android	Suicidality	3,50	3,00	4,25	3,17	3,60	2,67	2,63	3,00
WeCare, Fort Campbell	iOS	Suicidality	3,50	3,00	4,25	3,17	3,60	2,67	2,63	3,00
WeCare, Fort Campbell	Android	Suicidality	3,50	3,00	4,25	3,17	3,60	2,67	2,63	3,00
WeCare, Fort Detrick	iOS	Suicidality	3,50	3,00	4,25	3,17	3,60	2,67	2,63	3,00
WeCare, Fort Detrick	Android	Suicidality	3,50	3,00	4,25	3,17	3,60	2,67	2,63	3,00
WeCare, Fort Hood	Android	Suicidality	3,50	3,00	4,25	3,17	3,60	2,67	2,63	3,00
WeCare, Fort Jackson	iOS	Suicidality	3,50	3,00	4,25	3,17	3,60	2,67	2,63	3,00
WeCare, Fort Rucker	iOS	Suicidality	3,50	3,00	4,25	3,17	3,60	2,67	2,63	3,00
WeCare, Ft Bragg	iOS	Suicidality	3,50	3,00	4,25	3,17	3,60	2,67	2,63	3,00
WeCare, GA NG	Android	Suicidality	3,50	3,00	4,25	3,17	3,60	2,67	2,63	3,00
WeCare, GA NG	iOS	Suicidality	3,50	3,00	4,25	3,17	3,60	2,67	2,63	3,00
WeCare, JBSA	iOS	Suicidality	3,50	3,00	4,25	3,17	3,60	2,67	2,63	3,00
WeCare, JBSA	Android	Suicidality	3,50	3,00	4,25	3,17	3,60	2,67	2,63	3,00
WeCare, JRTC & Fort Polk	Android	Suicidality	3,50	3,00	4,25	3,17	3,60	2,67	2,63	3,00
WeCare, Resolute Support	Android	Suicidality	3,50	3,00	4,25	3,17	3,60	2,67	2,63	3,00
WeCare, USACE	iOS	Suicidality	3,50	3,00	4,25	3,17	3,60	2,67	2,63	3,00
WeCare, USACE	Android	Suicidality	3,50	3,00	4,25	3,17	3,60	2,67	2,63	3,00
WeCare, USAJFKSWCS	Android	Suicidality	3,50	3,00	4,25	3,17	3,60	2,67	2,63	3,00
WeCare, USAJFKSWCS	iOS	Suicidality	3,50	3,00	4,25	3,17	3,60	2,67	2,63	3,00
WeCare, USAREC	iOS	Suicidality	3,50	3,00	4,25	3,17	3,60	2,67	2,63	3,00
WeCare, 7th TB (X)	iOS	Suicidality	3,50	3,00	4,13	3,17	3,70	2,67	2,50	3,08
Coaches Assistance Program	Android	Suicidality	3,50	2,70	4,25	3,33	3,70	2,67	2,75	2,75
Self-Heal	iOS	NSSI	3,49	3,00	4,00	3,33	3,63	3,00	2,75	3,58
Suicide? Help! Tayside	iOS	Suicidality	3,49	3,20	4,13	3,33	3,30	2,50	2,75	2,92
trustTalk247	iOS	Suicidality	3,49	3,60	3,75	3,33	3,27	2,38	2,63	3,00
Ulster County SPEAK	iOS	Suicidality	3,49	3,30	4,13	3,17	3,35	2,67	2,50	2,92
STOPP app	Android	NSSI	3,48	2,80	4,50	3,50	3,13	2,83	2,13	2,33
oscER Jr. San Diego	iOS	Suicidality	3,47	2,60	4,25	3,50	3,55	2,50	2,50	2,75
oscER San Diego	iOS	Suicidality	3,47	2,60	4,25	3,50	3,55	2,50	2,50	2,75

WeCare, CASCOM	Android	Suicidality	3,47	3,00	4,13	3,17	3,60	2,50	2,25	2,75
SPEAK North Alabama	iOS	Suicidality	3,47	3,10	4,25	3,33	3,20	2,67	2,50	2,67
Albany County HOPE	iOS	Suicidality	3,46	3,50	3,50	3,33	3,50	2,67	2,75	3,42
Elijah	iOS	Suicidality	3,45	3,10	3,88	3,33	3,50	2,33	2,50	2,42
SPEAK North Alabama	Android	Suicidality	3,44	3,10	4,13	3,33	3,20	2,67	2,50	2,67
Urgencias psiquiátricas	Android	Suicidality	3,43	2,80	4,50	3,00	3,40	2,75	2,75	2,83
WeCare CASCOM	iOS	Suicidality	3,42	2,80	4,13	3,17	3,60	2,50	2,25	2,75
iwontbesilent	Android	Suicidality	3,42	3,00	3,75	3,33	3,59	2,50	2,38	2,84
Be Safe	Android	Suicidality	3,38	3,70	3,88	3,33	2,62	2,50	3,25	3,33
WeCare, JRTC & Fort Polk	iOS	Suicidality	3,38	3,00	4,25	3,17	3,10	2,67	2,63	3,00
Jason Foundation A Friend Ask	iOS	Suicidality	3,37	2,50	4,38	3,50	3,12	2,83	2,75	2,83
A.L.E.R.T.	iOS	Suicidality	3,37	3,40	3,38	3,50	3,20	2,67	2,75	3,33
Am I? Safety Plan	Android	Suicidality	3,35	3,10	3,63	3,33	3,33	2,88	3,00	3,50
Operation Reach Out	iOS	Suicidality	3,34	2,70	4,13	3,17	3,38	3,00	2,50	2,83
Jason Foundation A Friend Ask	Android	Suicidality	3,33	2,50	4,38	3,33	3,12	2,83	2,75	2,83
Coping Skills	iOS	NSSI	3,33	3,10	3,75	3,33	3,13	3,00	2,50	2,25
Don't panic - Depression and panic help	Android	Suicidality/NSSI	3,31	3,50	3,81	3,09	2,84	2,42	2,32	2,63
Step Up and Speak Out	iOS	Suicidality	3,31	2,60	4,13	3,00	3,50	2,67	2,50	2,83
Step Up and Speak Out	Android	Suicidality	3,31	2,60	4,13	3,00	3,50	2,67	2,50	2,83
Guard Your Buddy - Tennessee	iOS	Suicidality	3,29	2,40	4,13	3,33	3,30	2,33	2,50	2,84
Be Safe	iOS	Suicidality	3,29	3,70	3,50	3,33	2,62	2,50	3,25	3,33
HELP App-Prevention Resources	Android	NSSI	3,27	1,80	4,38	3,50	3,42	2,50	1,75	2,25
WeCare 59th Ordnance Brigade	iOS	Suicidality	3,27	2,50	4,13	3,17	3,30	2,50	2,38	2,50
WeCare, 59th Ordnance BDE	Android	Suicidality	3,27	2,50	4,13	3,17	3,30	2,50	2,38	2,50
Shatter the Silence	Android	Suicidality	3,26	2,30	4,13	3,33	3,30	2,33	2,25	2,50
MS DMH - Shatter the Silence	iOS	Suicidality	3,26	2,30	4,13	3,33	3,30	2,67	2,25	2,50
Hywel Dda Self Help Guides	Android	NSSI	3,23	2,20	4,00	2,83	3,88	3,17	2,13	3,25
Operation Reach Out	Android	Suicidality	3,22	2,50	4,00	3,00	3,38	3,00	2,50	2,83
The LifeLine	iOS	Suicidality	3,22	3,10	3,50	3,17	3,10	2,50	2,63	2,75
The LifeLine	Android	Suicidality	3,22	3,10	3,50	3,17	3,10	2,50	2,63	2,75
CerboCare: OCD and Depression Relief Self Help	Android	Suicidality	3,20	2,30	4,00	3,17	3,33	2,67	2,75	3,34
Community Stress First Aid	iOS	Suicidality	3,18	2,50	4,25	3,00	2,98	2,33	1,75	2,33
Alachua Talk	Android	Suicidality	3,18	2,50	3,63	3,00	3,60	2,58	2,63	2,75
MSE&SUICIDE ASSESSr	Android	Suicidality	3,18	3,30	3,75	2,67	3,00	2,63	2,50	2,25
Is Someone Suicidal?	Android	Suicidality	3,18	2,80	3,75	2,83	3,34	2,67	2,25	2,75
TUFMINDS	Android	Suicidality	3,18	3,40	3,63	2,83	2,85	2,67	2,00	2,67
eDIVO	Android	Suicidality	3,15	2,40	3,38	3,33	3,50	2,50	2,13	2,25
Suicide Lifeguard	Android	Suicidality	3,15	2,30	4,00	3,00	3,30	2,50	2,13	2,58
HELP Prevent Suicide	Android	Suicidality	3,14	2,30	4,00	3,33	2,92	2,50	2,25	2,75
HELP Prevent Suicide	iOS	Suicidality	3,14	2,30	4,00	3,33	2,92	2,29	2,46	2,75
Emotional Support Helpline Directory	Android	Suicidality	3,12	2,60	3,88	3,00	3,00	2,33	1,75	2,08
Prevención del suicidio	iOS	Suicidality	3,08	2,60	3,50	3,00	3,20	2,33	2,50	2,50
Self Harm Recovery	Android	NSSI	3,08	2,80	4,00	2,67	2,83	2,50	2,00	2,08

First Step Oregon	iOS	Suicidality	3,04	2,70	4,00	3,17	2,29	2,17	2,38	3,08
Suicidio y Psicología	Android	Suicidality	2,96	2,20	3,75	2,67	3,20	2,33	2,00	2,17
Self Harm Recovery	iOS	NSSI	2,86	2,60	3,50	2,50	2,83	2,29	1,75	1,92
Suicide Prevention -Ways to Help a Suicidal Friend	Android	Suicidality	2,76	2,60	3,63	2,17	2,64	2,17	1,75	2,34
PMCS Combating Suicide	iOS	Suicidality	2,75	2,30	3,75	2,17	2,80	2,17	1,75	2,33
Safe Hearts	iOS	Suicidality	2,72	2,30	3,13	2,67	2,80	2,00	1,63	2,50
A Teen Suicide Prevention Anime	Android	Suicidality	2,70	2,30	4,00	2,00	2,50	2,67	1,88	2,67
Alaska Careline	Android	Suicidality	2,52	2,50	2,50	2,33	2,75	2,46	2,25	2,67
Army Leader Smart Cards	Android	Suicidality	2,48	1,70	3,50	2,00	2,70	2,33	2,13	2,50
Mental Health - psychologist	Android	NSSI	2,35	1,90	2,88	2,33	2,28	2,17	1,38	1,58
Botón Anti-Suicidio	Android	Suicidality	1,88	1,00	4,00	1,00	1,50	2,33	1,50	0,67

1.4 Appendix D: Mean scores, Features and Functions of High-Quality MHA

Supplementary Table 5. Mean rating scores, features and functions of the high-quality MHA for suicide/NSSI prevention (full version)

Name	Pathology	Platform	MARS mean score	Target Group	Security and privacy	Evidence and certification	Credible Source
BackUp	Suicidality	iOS	4.53	AP<br>AE	PS, CI	WAT Label	✓
Calm Harm – manages self-harm	NSSI	iOS/Android	4.43	AP	PW, PS, CI	Award winning	✓
Better Stop Suicide	Suicidality	Android	4.4	AP	PS, CI	Award winning	✓
AuxiliaApp	Suicidality	iOS/Android	4.32	AP<br>AE<br>P	PW, PS, CI	Web/Aplicación de Psiquiatría-Psicología Acreditada	✓
Krisen Kompass	Suicidality	iOS/Android	4.31	AP<br>AE<br>B	PS, CI		✓
Friend2Friend	Suicidality	iOS	4.26	AE	PS,		✓
distrAct*	Suicidality/ NSSI	iOS/Android	4.24	AP	PS, CI	Certified member of the information standard	✓
mhGAP-IG 2.0 App (e-mhGAP)	Suicidality	iOS	4.2	P	PS, CI		✓
Be Safe	Suicidality	iOS	4.19	AP	PS, CI		✓
DMHS: Interactive Suicide Prevention	Suicidality	iOS	4.16	AP	CI		✓
Vrag Maar	Suicidality	iOS/Android	4.14	AP<br>AE	PS, CI		✓
Stay Alive	Suicidality	iOS/Android	4.11	AP<br>AE	PS, CI	Award winning	✓
Operation Life	Suicidality	iOS/Android	4.06	AP	PS, CI		✓
Suicide Prevention App	Suicidality	iOS/Android	4.06	AP<br>AE<br>P	PS, CI		✓
Jewish Care	Suicidality	Android	3.92	AP	PS, CI		✓
Prevent Suicide	Suicidality	iOS	3.89	AP<br>AE	PS, CI	Award winning	✓
Kokua Life	Suicidality	iOS	3.87	AP<br>AE	CI		✓
MoodTools - Depression Aid	Suicidality	iOS/Android	3.87	AP	PS, CI		✓
ReMinder Suicide Safety Plan	Suicidality	iOS/Android	3.87	AP	PS, CI		✓
TechSafe – Mental Health	NSSI	iOS/Android	3.86	AP<br>AE	PS, CI		✓
OnTrackAgain	Suicidality	iOS/Android	3.83	AP	PS, CI		✓
Prevent Suicide D&G	Suicidality	iOS	3.82	AP<br>AE	PS, CI		✓

Prevent Suicide - Highland	Suicidality	iOS/Android	3.82	AP<br>AE	PS, CI		✓
Prevent Suicide - NE Scotland	Suicidality	Android	3.81	AP<br>AE	PS, CI		✓
Prevensuic	Suicidality	iOS/Android	3.81	AP<br>AE<br>P	PS, CI		✓
Elevate Client	Suicidality	Android	3.8	AP<br>P	PS, CI		
Christian Veterinary Mission	Suicidality	Android	3.79	AP<br>AE	PS, CI		✓
Suïcide Preventie App	Suicidality	iOS/Android	3.79	P	PS, CI		✓
Cleveland State Univ Reach Out	Suicidality	iOS	3.78	AP<br>AE	PS, CI		✓
The Daily Difference	NSSI	Android	3.78	AP	PS		
I live	Suicidality	Android	3.77	AP	PS, CI		
Suicide Safety Plan	Suicidality	iOS/Android	3.76	AP	PS, CI		✓
Brothers for Life	Suicidality	iOS	3.75	AP	CI		
Student Health App	NSSI	Android	3.75	AP<br>AE	PS, CI	Certified member of the information standard	✓
DMHS: Suicide Prevention Info	Suicidality	Android	3.72	AP<br>AE	PS, CI		✓
Carpe Diem - Depression and Anxiety Forum	Suicidality	Android	3.71	?	?	?	?
SafetyNet: Your Suicide Prevention App	Suicidality	Android	3.71	AP	PS		
Columbus State Hope & Help	Suicidality	iOS	3.69	AP<br>AE	PS, CI		✓
MYPLAN – Your safety plan	Suicidality	Android	3.69	AP	PS	RCT protocol	✓
Lakeland Reach Out	Suicidality	iOS	3.69	AP<br>AE	PS, CI		✓
Students Against Violence MS	NSSI	iOS	3.68	AP<br>AE	PS, CI		✓
Backup Buddy (SCOT)	NSSI	Android	3.68	AP	PS, CI		✓
DMHS Suicide Prevention and Crisis Access Linkage Line (C.A.L.L.)	Suicidality	iOS	3.68	AP<br>AE	PS, CI		✓
Tri-C Help is Here	Suicidality	iOS	3.66	AP<br>AE	PS, CI		✓
Backup Buddy (SECAMB)	NSSI	Android	3.66	AP	PS, CI		✓
Gaia Teen Mind	NSSI	Android	3.66	AP	PW		✓

AP=Affected Persons; AE=Affiliated Environment; P=Professionals; PS=Privacy Policy; CI=Contact Information; PW = Password Protection; RCT= Randomized Controlled Trial

**According to the calculation of the mean score from suicide and NSSI rating*

1.5 Appendix E: Suicide Prevention Tools

Supplementary Table 6. Purpose and suicide/NSSI prevention strategies of high-quality MHA (full version)

	Purpose			Screening strategies		Accessing support strategies				Mental Health/treatment strategies					
	Information	Resources (emergency)	Urge/ behavior management	Physician-screening	Selfscreening	Peer and Family Support	Non-crisis Support	Crisis Support/	Visibility at all times	Psychotherapy	Safety Plan	Limiting access to means	Identification of<br>warning signs	 triggers	Coping strategies
BackUp	suicide	✓	suicide			✓		✓	✓	Information/ education<br>Tips/advice<br>Strategies/ skills<br>Urge management	✓		✓	✓	Relaxation<br>Exercise<br>Comfort<br>Distraction<br>Resource orientation<br>Mindfulness<br>Acceptance<br>Positive Reminders
Calm Harm – manages selfharm			NSSI		✓	✓				Tips/advice<br>Strategies/ skills<br>Urge management					Relaxation<br>Breathing<br>Exercise<br>Comfort<br>Distraction<br>Self-Expression<br>Resource orientation<br>Mindfulness<br>Acceptance
Better Stop Suicide			suicide		✓	✓		✓<br>(SC)		Information/ education					Relaxation<br>Breathing

									Tips/advice<br>Strategies/ skills<br>Urge management					Exercise<br>Distraction<br>Resource orientation<br>Mindfulness<br>Acceptance
AuxiliaApp	suicide	✓	suicide	✓	✓	✓		✓	Information/ education<br>Strategies/ skills<br>Urge management	✓				Resource orientation<br>Positive reminders
Krisen Kompass	suicide	✓	suicide		✓	✓	✓	✓	Information/ education<br>Tips/advice<br>Strategies/ skills<br>Urge management	✓	✓	✓	✓	Relaxation<br>Breathing<br>Resource orientation<br>Mindfulness<br>Acceptance<br>Mental Training
Friend2Friend	suicide	✓				✓		✓<br>(OH)	Information/ education<br>Tips/advice<br>Strategies/ skills			✓		Resource orientation
distrACT	suicide<br>NSSI	✓	suicide<br>NSSI			✓	✓	✓	Information/ education<br>Tips/advice<br>Strategies/ skills<br>Urge management			✓		Distraction<br>Resource orientation<br>Acceptance
mhGAPIG 2.0 App (emhGAP)*	Suicide<br>NSSI<br>Additional: Assessment tool	✓	Suicide<br>NSSI	✓		✓	✓	✓	Professional Assessment<br>Information/ education<br>Tips/advice		✓	✓		

Be Safe	✓	✓	suicide	✓	✓	✓	✓<br>(CAN)		Tips/advice<br>Strategies/<br>skills<br>Urge<br>management	✓			✓	Resource<br>orientation<br>Distraction<br>Comfort
DMHS:<br>Interactive<br>Suicide<br>Prevention	suicide	✓		✓	✓	✓	✓<br>(ON)	✓	Information/<br>education<br>Problem<br>Solving<br>Mood<br>Tracking	✓		✓		Relaxation<br>Breathing<br>Exercise<br>Comfort<br>Distraction<br>Self-<br>Expression<br>Resource<br>orientation
Vrag Maar	suicide	✓	suicide		✓		✓<br>(NLD)	✓	Information/<br>education<br>Tips/advice<br>Strategies/<br>skills<br>Urge<br>management	✓		✓		Resource<br>orientation<br>Positive<br>Reminders<br>Distraction<br>Comfort
Stay Alive	suicide	✓	suicide		✓	✓	✓<br>(UK)		Information/<br>education<br>Tips/advice<br>Strategies/<br>skills<br>Urge<br>management	✓	✓	✓		Relaxation<br>Breathing<br>Comfort<br>Resource<br>orientation<br>Mindfulne<br>ss<br>Grounding
Operation<br>Life		✓	suicide		✓	✓	✓ (AU<br>S)	✓	Tips/advice<br>Strategies/<br>skills<br>Urge<br>management			✓		Grounding<br>Mental<br>Training<br>Positive<br>Reminders<br>Resource<br>orientation
Suicide	suicide	✓		✓	✓	✓	✓			✓	✓	✓	✓	

Prevention<br>App	Additional: Assessment tool						(USA)		Information/<br>education<br>Tips/advice<br>Strategies/<br>skills<br>Urge<br>management<br>Professional<br>Assessment					Resource<br>orientation
Jewish Care	suicide	✓	suicide		✓	✓	✓<br>(AUS)	✓	Information/<br>education<br>Tips/advice<br>Urge<br>management	✓	✓	✓	✓	Positive<br>Reminders<br>Resource<br>orientation
Prevent<br>Suicide	suicide	✓	suicide		✓	✓	✓<br>(SCT)	✓	Information/<br>education<br>Tips/advice<br>Urge<br>management<br>Strategies/<br>skills	✓		✓	✓	Positive<br>Reminders<br>Resource<br>orientation<br>Distraction
Kokua Life	suicide	✓	suicide	✓	✓	✓	✓<br>(USA)		Information/<br>education<br>Tips/advice<br>Urge<br>management	✓		✓	✓	Positive<br>Reminders<br>Resource<br>orientation<br>Distraction
MoodTools<br>Depression<br>Aid*	✓	✓	suicide	✓	✓				Information/<br>education<br>Tips/advice<br>Urge<br>management	✓	✓	✓	✓	Positive<br>Reminders<br>Resource<br>orientation<br>Distraction<br>Relaxation<br>Breathing<br>Exercise<br>Mindfulne<br>ss
ReMinder<br>Suicide<br>Safety Plan		✓	suicide	✓	✓	✓	✓<br>(AUS)	✓	Tips/advice<br>Strategies/<br>skills<br>Urge<br>management	✓	✓	✓		Relaxation<br>Breathing<br>Resource<br>orientation

TechSafe Mental Health	NSSI	✓		✓	✓			Information/ education Tips/advice				Resource orientation
OnTrackAgain		✓	suicide	✓		✓ (NLD)		Tips/advice Strategies/ skills Urge management	✓	✓	✓	Resource Orientation Relaxation Mindfulness Positive Reminders Distraction
Prevent Suicide D&G	suicide	✓	suicide	✓	✓	✓ (SCT)	✓	Information/ education Tips/advice Urge management Strategies/ skills	✓	✓	✓	Positive Reminders Resource orientation Distraction
Prevent Suicide Highland	suicide	✓	suicide	✓	✓	✓ (SCT)	✓	Information/ education Tips/advice Urge management Strategies/ skills	✓	✓	✓	Positive Reminders Resource orientation Distraction
Prevent Suicide NE Scotland	suicide	✓	suicide	✓	✓	✓ (SCT)	✓	Information/ education Tips/advice Urge management Strategies/ skills	✓	✓	✓	Positive Reminders Resource orientation Distraction
Prevensuic	suicide	✓	suicide	✓		✓		Information/ education Tips/advice Strategies/ skills Urge management	✓	✓		Resource Orientation Positive Reminders Distraction

Elevate Client	Additional: Connection with therapist		✓	✓	✓	✓	✓		Strategies/<br>skills	✓	✓	✓	✓	Mindfulness<br>Distraction<br>Resource orientation
Christian Veterinary Mission	suicide	✓				✓	✓<br>(USA)		Information/<br>education<br>Tips/advice		✓			Resource orientation
Suïcide Preventie App	suicide								Information<br>education<br>Tips/advice<br>Strategies/<br>skills			✓		Resource orientation
Cleveland State Univ Reach Out	suicide	✓			✓	✓	✓<br>(USA)	✓	Information/<br>education<br>Tips/advice<br>Strategies/<br>skills			✓		Resource orientation
The Daily Difference	Suicide<br>NSSI	✓	Suicide<br>NSSI	✓					Information/<br>education<br>Tips/advice<br>Urge management			✓	✓	Relaxation<br>Breathing<br>Reflection<br>Exercise<br>Comfort<br>Distraction<br>Self-Expression<br>Mindfulness
I live		✓	✓		✓	✓	✓	✓	Tips/advice<br>Strategies/<br>skills<br>Urge management	✓			✓	Relaxation<br>Breathing<br>Resource orientation<br>Positive Reminders<br>Grounding
Suicide Safety Plan	suicide	✓	suicide		✓	✓	✓<br>(USA)		Information/<br>education<br>Tips/advice<br>Urge management	✓	✓	✓	✓	Positive Reminders<br>Resource orientation<br>Distraction

Brothers for Life	*	*	*	*	*	*	*	*	*	*	*	*	*	*	*
Student Health App	NSSI	✓				✓	✓			Information/ education<br>Tips/advice			✓		Resource orientation<br>Distraction<br>Self-Expression
DMHS: Suicide Prevention Info	suicide	✓	suicide		✓	✓	✓	✓ (CAN)	✓	Information/ education<br>Tips/advice<br>Strategies/ skills<br>Urge management	✓	✓			Resource orientation<br>Grounding<br>Distraction<br>Mindfulness
Carpe Diem Depression and Anxiety Forum															
SafetyNet: Your Suicide Prevention App		✓	suicide		✓	✓		✓	✓	Tips/advice<br>Urge management<br>Strategies/ skills	✓			✓	Relaxation<br>Breathing Exercise<br>Self-Expression<br>Resource orientation<br>Distraction<br>Grounding
Columbus State Hope & Help	suicide	✓				✓	✓	✓ (USA)	✓	Information/ education<br>Tips/advice<br>Strategies/ skills			✓		Resource orientation
MYPLAN – Your safety plan	✓	✓	✓			✓	✓	✓		Information/ education	✓		✓		Resource orientation

									Strategies/<br>skills<br>Urge<br>management				
Lakeland<br>Reach Out	suicide	✓			✓	✓	✓<br>(USA)	✓	Information/<br>education<br>Tips/advice<br>Strategies/<br>skills			✓	Resource<br>orientation
Students<br>Against<br>Violence MS	Suicide<br>NSSI	✓			✓	✓	✓<br>(USA)		Information/<br>education<br>Tips/advice				Resource<br>orientation
Backup<br>Buddy<br>(SCOT)	NSSI	✓			✓	✓	✓<br>(UK)		Information/<br>education<br>Tips/advice				Distraction<br>Comfort<br>Grounding<br>Resource<br>orientation
DMHS<br>Suicide<br>Prevention<br>and Crisis<br>Access<br>Linkage Line<br>(C.A.L.L.)	suicide	✓	suicide	✓	✓	✓	✓<br>(CAN)	✓	Information/<br>education<br>Tips/advice<br>Strategies/<br>skills<br>Urge<br>management	✓	✓		Resource<br>orientation<br>Grounding<br>Distraction<br>Mindfulne<br>ss
TriC Help is<br>Here	suicide	✓			✓	✓	✓<br>(USA)	✓	Information/<br>education<br>Tips/advice<br>Strategies/<br>skills			✓	Resource<br>orientation
Backup<br>Buddy<br>(SECAMB)	NSSI	✓			✓	✓	✓<br>(UK)		Information/<br>education<br>Tips/advice				Distraction<br>Comfort<br>Grounding<br>Resource<br>orientation
Gaia Teen<br>Mind	NSSI	✓	NSSI	✓	✓	✓			Information/<br>education<br>Tips/advice<br>Strategies/<br>skills				Resource<br>orientation<br>Self-<br>Expression<br>Relaxation

Urge management	Comfort<br>Exercise<br>Distraction<br>Grounding

SC=Several Countries; OH=Ohio; CAN=Canada, ON=Ontario; NLD=Netherlands; UK=United Kingdom; AUS=Australia; SCT=Scotland; USA=United States of America *Unavailable at the time of assessment
